# Proceedings of the American Dermatological Association

**Published:** 1877-10

**Authors:** 


### Reports of Societies.

### PROCEEDINGS OF THE AMERICAN DERMATOLOGICAL ASSOCIATION.—FIRST ANNUAL MEETING.*

The Association was called to order at 10 A. M., Sept. 4th, in the Club Rooms of the Cataract House, Niagara Falls, by the President, Prof. James C. White, of Boston. The Report of the Council was read by the Secretary, Dr. L. Duncan Bulkley, of New York. Several names were then proposed for active and honorary membership; and an auditing committee appointed, consisting of Drs. Wm. Brodie, of Detroit, and I. E. Atkinson, of Baltimore.

The President then read his annual address, which contained an interesting historical sketch of the progress of dermatology in America, an eloquent tribute being paid to the labors and teachings of Prof. Hebra, of Vienna, and to their influence especially upon American students abroad. It was in consequence of these teachings that dermatologists had learned to observe diseases of the skin, just as naturalists learn to observe; and, by the aid of the abundant material in dispensaries and hospitals, studied in accordance with the new and better method, it had come to pass, finally, that the men who once shone with reflected light, are now themselves contributors. Looking at the character of the instruction given in dermatology in American medical schools twenty-five years ago, we find a decided improvement noticeable in 1871, when, though no American treatises had been published on the subject, still there was much work and observation, and a promise of better things. To-day we have, in many of the schools, special instruction given, and the only journal specially devoted to dermatology in the English language, is published in this country. He (the president) had received 39 replies to a

* In the compilation of this report, I have simply enlarged the excellent report o Dr. Edw. Wigglesworth, (published in the Boston Med. and Surg. Journal, Sept. 13, 1877,) by the addition of notes taken by myself during the sessions.—J. N. H.

circular issued by himself, asking for the number and title of published contributions, and the journals in which they have appeared. These thirty-nine replies reported 258 contributions on dermatological subjects, and 54 on subjects connected with syphilis; in all 312. There were also translations, reviews, analyses; two special treatises by American authors; one atlas. The success of the N. Y. Dermatological Society had undoubtedly contributed to the result.

The objects of the Association are: 1. The affording of opportunities for a more intimate personal acquaintance among American dermatologists, thus obviating the tendency to harsh judgments upon necessary differences through ignorance of personalities. 2. To average the varying views upon ætiology and treatment, by oral discussion in each others' presence, and thus to elaborate a common basis for work. 3. To ascertain by observant study and by the collection of statistical data, the peculiarities of cutaneous diseases at present existing in this country, the establishment of a standing committee for this purpose being desirable, and to investigate new diseases, such as leprosy, which seems to have made its appearance in the South. 4. The establishment of a common nomenclature in dermatology. 5. To foster the interests of dermatology in its general relations to the medical profession and to the public.

The objects of the Association show the need of its existence. There are peculiar difficulties and very inappropriate rewards in this specialty of medicine. The medical profession has yet to learn that diseases of the skin are like other diseases, all being subject to the same laws, and all affecting organs composed of similar tissues. Physicians either lack interest in these maladies, or, regarding them as incurable, leave them to chance; or, again, pandering to popular prejudice, talk of "humors," and of diseases "coming out," or "striking in," thus frightening patients into resignation; or will unjustifiably undertake the treatment of maladies of which they are ignorant, merely because they anticipate less evil from mismanagement than they would in the care of maladies of the eye and ear, for instance. Even physicians who consult a specialist, seem surprised that these diseases subsequently remain apparently as intractable as before, forgetting that the pathological conditions may vary from day to day in an organ so exposed, thus calling for a coincident change, perhaps temporary only, in the plan of treatment. There is a necessity for special teachers, for purposes of education in medical schools. There exists the need of better opportunities for instruction by means of special clinics, either in special hospitals, or in special wards in established institutions. The neglect of existing institutions to do common justice to the patient suffering from these diseases, is an outrage against reason and humanity.

On motion of Dr. Bulkley, a committee was appointed to consider and report upon the recommendations contained in the address, consisting of Dr. Heitzmann, of New York, Dr. Hardaway, of St. Louis, and Dr. Wigglesworth, of Boston.

A paper upon Acute Conditions of Disease excited by the iodide of potassium, by Dr. A. Brooks, of Chicago, was then read out of turn. The writer called attention in it, not to the various cutaneous lesions which the drug is capable of producing, but to varieties of arthritis, iritis and fever, which he believed, were sometimes induced by it.

Dr. I. E. Atkinson had seen affections of several of the cranial nerves induced by the drug, and in one case, erythema of the buccal mucous membrane. He had given two drachms ter die for a month, in conjunction with the biniodide of mercury.

Dr. R. W. Taylor had seen acne, arthralgia, with pain in the sclerotica and conjuctivitis, follow even small doses; after large doses, he had noticed only dermatic lesions, even bloody pemphigus, but without rise in temperature. He had given, by degrees, as much as twelve drachms per diem, for many days; in one case, 20 drachms for a sciatica due to syphilis, which amount, however, produced less effect than 14 drachms combined with two drachms of the bromide of potassium.

Dr. Hardaway had seen syphilitic albuminuria disappear under similar doses.

Dr. L. A. Duhring regretted that the paper had not discussed the cutaneous lesions induced by the drug, and narrated the case of a lad, who applied for treatment of an oozing and crusted patch of infiltrated eczema upon the fore-arm. There was also a vesicular and bullous eruption upon the wrists, fore-arms, backs and palms of the hands. The vesicles were from pin-head to pea-sized, elevated and in places confluent, resembling variola, though there were no pustules. The whole picture was a *fac simile* of Tilbury Fox's plate of dysidrosis. The eruption appeared the day after taking three or four ten grain doses of the iodide. The vesicles oozed when punctured, but did not collapse, filled as they were by a gelatiniform secretion, exactly resembling a grain of boiled sago, when viewed from without. There was no itching, œdema, nor febrile disturbance. The eruption dried up in a week, without treatment. The eczema was not influenced by the drug.

Dr. Van Harlingen had also seen the case and added that the eruption was essentially upon the extensor surfaces, becoming more scattered as it extended up the arm. The ezcematous patch was about 4 by 3 inches, and upon the flexor surface. The vesicles were umbilicated. The pulse quite normal. After the symptoms had disappeared, the drug was again administered for the sake of experiment, and the vesicles immediately re-appeared.

Dr. J. N. Hyde had given one and one half drachms *ter die* for a week in one case, and added that the writer of the paper under discussion, had stated in his presence, that he had administered one thousand grains *per diem.* The speaker referred to the fact that often a small or moderate dose of the iodide, would produce the phenomena of coryza, &c., while the larger doses mentioned, were occasionally very well tolerated.

Dr. W. A. Hardaway had seen intense urticaria, even purpura urticata upon a tuberculous patient, who had taken half a grain of the iodide.

The first paper of the afternoon session was read by Dr. L. D. Bulkley of New York, and entitled, On the so-called Eczema Marginatum of Hebra (tinea circinata cruris) as observed in

America [1]. Dr. Bulkley's treatment was by sulphurous acid.

Dr. Heitzmann considered the disease the same in this country as in Vienna. He did not consider the success of the treatment, so far as time was concerned, any greater than by other methods. He had repeatedly cured cases in a week by means of Wilkinson's ointment spread upon rags and closely and continuously applied.

Dr. Duhring remarked that Eczema might be found in the region attacked by the so-called Eczema Marginatum, and should bear its own name. That tinea circinata might also exist there and ought to retain its own name; that, furthermore, the two diseases were, from the first, distinguishable from each other. In Philadelphia the disease assumed a milder type.

Dr. R. W. Taylor remarked that he had observed one of the cases reported, which certainly seemed to be a marginate form of eczema, merely. The itching was severe, the margin was defined by glazed papules, the affected integument was of a violaceous brown color, and there was coexistent eczema manuum. He had not discovered the parasite.

The president remarked that he recognized three distinct varieties of disease in this locality, having the general features described. 1. There was true ringworm—the development of the *tinea tricophytina* being the same here as in Europe. 2. Chronic and advanced cases, where the two diseases were combined, the eczema becoming subsequently inoculated with the parasite, or the development of the latter inducing an eczema, as it would in patients subject to this affection. He had seen cases of "Burmese Ringworm" where, after destruction of the parasite, the eczema only died out very gradually. 3. Cases of eczema, where no parasite existed. The margin of the affected patches was defined by an elevated border. There was no resemblance here to either tinea favosa or tinea versicolor. The disease is slow of cure, and although Wilkinson's ointment is very valuable, he had never obtained from it the speedy effects claimed by Dr. Heitzmann.

A paper on the Pathology of Seborrhœa was then read by

(1) This paper will appear in full in the November No. of the Chicago Medical Journal and Examiner.

Arthur Van Harlingen, M. D., of Philadelphia. The following were the author's conclusions:

1. The sebaceous secretion is derived from fatty metamorphosis of the enchyma cells of the sebaceous glands. These cells are homologous with those of the stratum mucosum of the skin. They have nothing in common with the cells of the horny layer.

2. Seborrhœa is a disease of the sebaceous glands, characterized by the pouring out of an increased quantity of sebum, more or less altered in chemical and physical composition. In comedo and seborrhœa sicca, properly so called, the secretion is condensed to a fatty consistency, while in seborrhœa oleosa it remains in an oily state; in each of these affections, however, microscopic examination shows epithelial cells in a state of more or less complete fatty degeneration and breaking down into granular debris. Horny cells are only found adventitiously.

3. Certain forms of disease heretofore commonly classed as seborrhœa sicca, should properly be removed from the category of diseases of the sebaceous glands, since the pathological product in these cases is not sebum, but epithelium from the horny layer of the skin. Any sebum which may be present is a mere accompaniment of the epithelial product. For these cases the designation pityriasis, or pityriasis simplex would seem appropriate.

Dr. G. H. Fox referred to a case where the whole glans penis was enveloped as with smegma, by an apparently epithelial growth, which could be scaled off in laminæ.

The president spoke of this condition as one of a commencing horny growth, the development of epithelial matter being accompanied by enlargement of the papillæ, and followed by horn formations. For two years he had had such a case under observation while developing. At first laminæ could be removed.

Dr. G. H. Fox then read a paper upon molluscum contagiosum, based upon the observation by himself of twenty-six cases. Five per cent. of these occurred upon the male genitals. He believed the affection to be rare in private practice; and called attention to its frequent co-existence with warts; this co-existence being of such frequent occurrence that it is to be regarded as more than an accident. He suggested that the term molluscum simply, should be applied to the disease, and that the affection designated as *molluscum fibrosum* be termed fibroma, thus clearly distinguishing the two.

Dr. Heitzmann stated that he had examined two such cases under the microscope, and found epithelial elements in a state of fatty degeneration, containing the molluscous bodies ot Virchow and Rindfleisch, which are supposed to be the bearers of contagion. He had seen molluscum occurring upon the thighs of women after leucorrhœa, just as papillomatous warts may arise by contact with the secretion of blennorrhagia, of which he had seen one case upon the face. The sebaceous glands, the root sheaths of the hairs, etc., may be at times in such a condition that irritative stimulation is alone needed for the production of the new growth. Dr. Heitzmann recognized the relationship between molluscum and common warts, and did not regard the former as contagious. Recent investigations at Vienna also tend to disprove any contagiousness.

Dr. Hyde, referring to Dr. Heitzmann's remarks, stated that the vegetations induced by fluids of a blenorrhagic nature, did not necessarily result in a single individual from even the most virulent of secretions. It seemed as though the process of "acclimatization of the penis," first described by Ricord, often influenced the development of these growths. In certain cases an individual who has had several attacks of blennorrhagia, will find the most luxurious development of vegetations first occurring after contact with secretions, to which he is unaccustomed; and which are not nearly so acrid as those to which he had been formerly exposed.

Dr. Duhring spoke of the rarity of molluscum among the upper classes in Philadelphia, where, however, it did occur. He had rarely met with well developed cases in children, though he had seen scores where the appearances were ill developed, or in an abortive stage, the size perhaps of a pin's head.

Dr. E. Wigglesworth, of Boston, was also skeptical in regard to the contagiousness of the disease in question, although he could say, in answer to Dr. Duhring's remarks, that he himself had been at one time the subject of an attack of molluscum, occurring, curiously enough, but a short time subsequently to his having expressed with his thumb-nails the contents of several molluscous papules, for purposes of microscopical examination, from a patient in dispensary practice. In his own case the individual lesions had been found successively, and upon various parts of the trunks and limbs.

Dr. Wigglesworth's paper upon Faulty Innervation as a factor in Skin Disease, was then read by title only, owing to illness upon his part.

A short paper by Dr. Dyce Duckworth, of London, upon the Treatment of Severe Bed Sores, was next read by the secretary, and the Association adjourned.

SECOND DAY, SEPTEMBER FIFTH.

After the reading and acceptance of the Report of the Treasurer and Auditing Committee, the Report of the Committee on the President's Address was received and considered. In accordance with the reccommendations contained in the latter, the following committees were appointed: on *Nomenclature*: the President, *ex officio*, and Drs. Duhring, Taylor, Wigglesworth and Heitzmann; on *Statistics*: Drs. White, Bulkley, Hyde, Atkinson, Hardaway, Yandell, Van Harlingan and Brodie. On motion it was agreed that the bibliographical statement prepared by the President be incorporated with the transactions of the Association. The following officers were elected for the ensuing year:

*President*—James C. White, of Boston. *Vice-Presidents*—L. D. Bulkley and C. Heitzmann, of New York. *Secretary*—R. W. Taylor, of New York. *Treasurer*—I. E. Atkinson, of Baltimore.

The following were chosen honorary members of the Association by unanimous vote:

Prof. F. Hebra, of Vienna; Prof. Erasmus Wilson, of London, and Prof. Hardy, of Paris.

The following named gentlemen were chosen active members:

Dr. W. H. Geddings, of Aitken, S. C.; Dr. Silas Durkee, of Boston, Mass; Dr. F. P. Foster, of New York, and Dr. Sam'l. Sherwell, of Brooklyn, L. I.

Dr. Hyde announced a brief paper describing a "rare form of multiple, congenital and monolateral pigmentary nævus," and received permission to present it.

Dr. Campbell, of New York, then read a description of a Case of True Prurigo (of Hebra).

Dr. Duhring had seen this case, and but one one other in this country, a case shown him some four years ago by Dr. Wigglesworth, occurring in Boston. General discussion showed that none of those present had ever observed more than two cases, and these, for the most part, were the same cases seen by the various physicians.

Dr. Wigglesworth, in addition to the case alluded to by Dr. Duhring, the first case ever published in this country, reported in full in the January No. for 1873, of the *American Journal of Syphilography and Dermatology*, had seen another case occurring upon a farmer, where the formation of papules, the thickening of the skin, the buboes in the groins, the locality of the parts affected, the peculiar appearance to the eye, and to the fingers after rubbing them upon the lower limbs, the sound caused by this, the discoloration, furrows and scratch marks, were all symptomatic of prurigo. The only opposing evidence was the statement of the patient, that the disease did not begin in infancy. The case had, unfortunately, been lost sight of in spite of every caution.

The president had never seen a case of true prurigo in this country, and while, of course, not disputing the fact that such had been observed, still he thought that great care should be taken in not mistaking chronic cases of eczema with much infiltration of the skin and secondary changes, for true prurigo.

Dr. J. N. Hyde, of Chicago, then read a paper upon the Immunity of Certain Mothers of Children affected with Hereditary Syphilis. His conclusions were as follows :

1. That if the possibility of the occurrence of conception without maternal infection be admitted, it follows that direct infection of the wife by the husband may occur at any subsequent period of the gestation. Hence, the date of appearance of maternal syphilis cannot be urged in support of the so-called "syphilis by conception."

2. That, inasmuch as the blood of the husband is capable of transmitting the disease directly to his healthy wife, the non-contagious character of the lesions exhibited by the former, cannot be urged in favor of his innocuousness during the pregnancy of the latter.

3. That many of the physiological and pathological phenomena of pregnancy, render it highly improbable that syphilis of the mother should exist without external manifestations; there being, further, evidence of the fact, that puerperal and scarlet fevers and erysipelas in the human female, as well as spontaneous vaccinia and equinia, are contagious disorders, connected with, and often originating in, abnormal puerperal conditions.

4. That the mode of development of the fertilized ovum demonstrates the phase of its physiological independence of the maternal organism, the placenta discharging a respiratory function and presenting an effectual barrier against intra-uterine infection.

5. That there is evidence to show that not only trichinæ, but various other poisonous organisms, are incapable of transmission through the placental parietes; and that the proofs of such transmission in the case of the exanthematous fevers, and variola in particular, cannot be considered as fully established.

6. That the full weight of Colles' Law is to be estimated in connection with the question whether the child whose hereditary syphilis is derived from the mother exclusively, is capable of infecting its healthy father; and, if no evidence of this latter can be adduced, a higher law becomes defined; viz., that the child whose hereditary syphilis is transmitted by one parent only, is incapable of infecting either.

7. That, if such immunity be established, it is probably due to the fact that the syphilis-bearing cell element cannot readily be implanted upon the soil from which it sprang—a fact illustrated by the infecundity of consanguineous marriages, and the non-auto-inoculability, in general, of the primary lesion of syphilis.

Dr. R. W. Taylor remarked that his own view as to the main question was clearly expressed in his paper upon the subject. He thought that the immunity of the mother was due, not to her infection, but to an influence, resulting from her carrying a syphilitic child, which made her insusceptible to the action of the virus.

Dr. Hardaway called attention to the fact, recently shown by Fournier, that the law of Colles had a double bearing—establishing not only the immunity of the mother, but that also of the child.

Dr. Atkinson believed that the syphilitic virus affected the protoplasm itself, and that it was impossible for the interposition of a membrane to check its progress—as impossible as for any membrane of the body in acquired syphilis, to stay its advance. The discussion was then postponed to the afternoon.

A paper was then read by Dr. C. Heitzmann, of New York, on the relation of Impetigo Herpetiformis to Pemphigus, based upon a case observed by himself.

The first paper of the afternoon was by Dr. W. A. Hardaway, of St. Louis, upon the Lymphatic Theory of Syphilitic Infection, with a new view of the Relation between Chancre and Chancroid, and suggestions for the radical cure of syphilis.

Dr. Atkinson, (having had the floor when the debate upon Dr. Hyde's paper was suspended,) went on to say that since the syphilitic virus existed in blood corpuscles and in lymphatic and spermatic cells, wherever the protoplasm of the body reaches, there the virus may reach. He believed in the law of Colles, considering the mother as already infected, the wall between the mother and the fœtus having been already inoculated by the syphilitic protoplasm nourishing the fœtus. The speaker, like Dr. Hardaway, considered the lymphatic system the medium of contagion. He did not regard induration as the necessary sign of infection. All pus is contagious. When unformed, immature, or less virulent, its element might enter into the system, carrying the constitutional infection. Formed pus, although syphilitic, caused merely a local lesion, being too large to enter the blood. Could it do so, it would produce disease even more violent than syphilis.

Dr. Heitzmann declared his belief that at no distant day these problems would be all solved by the microscopist. He was now able to detect the difference between healthy blood and that of a scrofulous patient—merely by using his objectives.

Dr. Hyde stated that, the discussion having been prolonged, he would not occupy time further than to state that the question regarding the lymphatic system as that by which infectious material was introduced into the blood-mass, had passed from the domain of speculation and theory, and was an established and accepted proposition. Were other evidence wanting, the recently published experiment of Maurice Reynaud—reported by the latter to the French Academy,—was sufficient to settle the question. Reynaud produced horse-pox by inoculation, and when the vesicles were fully developed, he laid bare a lymphatic vessel passing from the site of the lesions, opened it, established a lymphatic fistula, collected the lymph, injected it into the jugular vein of another horse, and, after a due period of incubation, had the satisfaction of seeing the second animal covered with a superb eruption of horse-pox vesicles.

Dr. R. W. Taylor then read a paper on the Xeroderma of Hebra, being an exhaustive description, with comments, of seven cases of this rare form of disease, observed by himself in New York City. Photographs of these patients were exhibited to the Association, the larger number occurring in a single family of Austrian Jews, residents of New York.

Dr. Heitzmann had observed in Vienna two cases of this affection and subsequently in this country, upon an immigrant, the first case ever observed here, one which had been already described by Dr. Lewin, of Berlin. He had seen the four cases of Dr. Taylor, and since these two others, one being at present under treatment. This last is the case of a male adult, who has had the disease for thirty years. The patches exist upon the face and hands. There is also an ulceration upon the left cheek, which Dr. Heitzmann regarded at first as rodent ulcer, and upon which he has operated four times with a dermal curette or scraping spoon, the wound in each case healing kindly. Every six months, however, a nodule again forms. Microscopic examination showed the presence of the elements of epithelial cancer, agreeing with the description of Kaposi.

The president called attention to the resemblance of this disease in its early stages to the "morphœa" of Wilson, the erythema and pigmentary deposits being followed by flat atrophy of the skin. Cancerous growths may appear upon many forms of papillary hypertrophy or of pigmentary deposits in other skin diseases as well as in this.

Dr. Duhring spoke of the resemblance of these cases to morphœa or the keloid of Addison, so far as regards the teleangeiectasic spots over limited areas of different parts of the body, lasting perhaps a few years, and then undergoing spontaneous involution. The same category may ultimately include these two diseases as morphœa. He regarded the disease in question as an atrophy. In some cases of morphœa also there are marked changes in the capillaries as well as in regard to pigment.

The paper next presented was by Dr. L. P. Yandell, Jr., of Louisville, upon The Ætiology of Cutaneous Diseases.

In reply to Dr. Bulkley, the reader stated that he had found at times in old cases of eczema capitis in children that a periodicity existed in the pruritus, and that he had been able to relieve this by doses of quinine and other anti-periodics without other treatment; so also in cases of eczema upon the upper extremities. It was more difficult where the lower limbs were affected, especially if varicose veins existed. He regarded acute affections of the skin as due to malaria, chronic ones as arising from a scrofulous taint.

The president referred to the prevalence of acute skin diseases in places where malaria did not occur. The reader, no doubt, observed skin diseases upon patients already suffering from malaria, but in Eastern cities they occurred also, and where no malarious taint was in operation. There have been only three cases of primary malaria ever observed in Boston. Where malaria already exists it may be a predisposing cause. The question depends, then, upon the relative frequency of diseases of the skin in places where there is malaria and where this does not exist. It would be interesting if Dr. Yandell would furnish such statistics at the next annual meeting of the Association, and show the differences between skin diseases, as observed by him in malarious regions and those elsewhere.

Dr. Heitzmann said that in Hungary, where there is much malaria, a Dr. Poor had advanced this theory some ten years since; while in Vienna, where "scrofula" abounded, this last was regarded as the *fons et origo mali.* Both theories had at that time been disproved and the question settled.

The Association then adjourned.

THIRD DAY, SEPTEMBER SIXTH.

Dr. L. A. Duhring, of Philadelphia, read a Case of Undescribed Form of Fragilitas Crinium, in which the hairs were split throughout their whole length, from the bulb to the free extremity; and showed specimens under the microscope.

A paper upon Two Cases of very late Hereditary Syphilis was then read by the secretary, Dr. L. D. Bulkley, of New York.

Dr. Taylor thought that there was an immunity possessed by the skin after the age of twenty years, and he would distrust all symptoms occurring later than this period for the first time, and refer them rather to acquired syphilis.

Dr. Atkinson referred to a case of syphilis inherited through two generations, reported by him in the *Archives of Dermatology* for January, 1877.

The president had seen atrophy of the dental tissues occur in the second teeth, where no syphilis existed, the case being preceded by severe illness of an acutely inflammatory type. Here the lower teeth, the molars and canines were sound, the lateral upper incisors wanting, and the central ones notched.

Papers upon the Pathological History of Psoriasis, by Dr. A. R. Robinson, of New York; The Auto-Inoculation of Vegetable Parasites, and their Non-Identity, by Dr. E. Wigglesworth, of Boston; and Affections of the Testicle in Hereditary Syphilis, by Dr. R. W. Taylor, of New York, were then read by title only.

Dr. Van Harlingen showed tubes, like those used in oil painting but larger, for the purpose of preserving and dispensing ointments, which should be melted and poured into the open bases of the tubes, these being then closed. This offered also a cleanly method of transporting ointments. They were also in use for English shaving soaps, and could be obtained of all sizes, of Remington, 18th and Walnut streets, Philadelphia.

Dr. Fox had used these to inject ointments through a soft catheter into the deep urethra.

After the induction of the newly-elected officers, the Association adjourned to meet at Saratoga, New York, on the last Tuesday of August, 1878.

